# Is there a relationship between personal reflection ability and moral reasoning ability in Indonesian medical students?

**DOI:** 10.5116/ijme.5b64.971a

**Published:** 2018-08-17

**Authors:** Eti N. Sholikhah, Yoga P. Susani, Yayi S. Prabandari, Gandes R. Rahayu

**Affiliations:** 1 Faculty of Medicine, Public Health, and Nursing, Universitas Gadjah Mada, Yogyakarta, Indonesia; 2 Faculty of Medicine University of Mataram, Lombok, Indonesia

**Keywords:** Personal reflection, moral reasoning, medical student, the Defining Issues Test (DIT), the Groningen Reflection Ability Scale (GRAS)

## Abstract

**Objectives:**

To determine the relationship of personal reflection ability and moral reasoning ability of medical students of the Faculty of Medicine Universitas Gadjah Mada (UGM).

**Methods:**

A cross-sectional study was conducted by distributing questionnaires to 293 medical students in Year-3 at the Faculty of Medicine, Public Health, and Nursing after obtaining their agreement to participate in this research by signing an informed consent form. Personal reflection ability was measured by the Groningen Reflective Ability Scale (GRAS) questionnaire; moral reasoning ability was measured by the Defining Issues Test (DIT) questionnaire. Descriptive statistics, T-test, and regression analysis were used to analyze the anonymized results.

**Results:**

The mean GRAS score of all students was 89.59 (SD = 6.80) (GRAS score ranged 0-110) which showed a high level. The mean score of Principled Morality Score (P) DIT of all students was 32.39 (SD=11.04), ranging between 28-41 which indicated a moderate level. In general, personal reflection ability scores of all students were positively correlated with their moral reasoning ability score. However, this correlation was not significant statistically (r=0.080, p=0.239).

**Conclusions:**

Personal reflection ability of medical students was correlated positively with their moral reasoning ability, however, statistically it was not significant . The high level of personal reflection ability needs to be maintained. The moderate moral reasoning ability needs some efforts to improve it. Further studies are necessary to assess other factors which influence the moral reasoning ability so that appropriate interventions can be developed.

## Introduction

Personal reflection for medical students involves reflection on their experiences to emphasize the direction of reflective attention to the process of sense-making and decision-making in medical practice, and to the dynamics of rational and irrational thoughts and emotions, assumptions, and beliefs in that process. Personal reflection in the medical field can be described as the exploration and appraisal of an individual’s own and other’s experiences, thus clarifying and creating meaning, for the benefit of achieving a balanced functioning, learning, and development. Personal reflection integrates some key aspects: mindfulness rather than intellectualism; attention to experience rather than action; appropriate communication, such as handling a dialogue and feedback rather than debate and discussion; clarifying the process of sense-making rather than problem solving; and personal inner reflection resulting in transforming or confirming one’s own perspectives on professional practice and identity.^1^

Personal reflection is an important component of medical education. Individuals develop reflections, but the reflections of medical students should be guided so that the important foundations of medical beliefs and assumptions can be achieved. A reflection should be made individually because each person has a certain preferred approach. Although there is little evidence to suggest that the reflection process directly improves patient care, however, it still possibly will affect the treatment process.^2^ Personal reflection is an important activity in the learning process. In clinical medical education, personal reflection allows the students to connect the knowledge they have gained with experiences in dealing  with  patients  and  the  real-life situation in  clinical rotation.^3^ Mann et al. reported that the ability of reflection did not happen by itself, however, it can be stimulated by the educational process.^4^

Moral reasoning is not simply about deciding good or bad. Instead it is the way a person thinks and comes to the decision that something is good or bad. One theory of moral reasoning is Kohlberg's theory, according to which moral development is divided into three stages: the pre-conventional, conventional and post-conventional. Someone who is in the pre-conventional level tends to judge the morality of an action by its direct consequences. Someone at the stage of the conventional level judges the morality of an action by comparing it with the views and expectations of society. In the post-conventional level, individual judgment is based on self-chosen principles, and moral reasoning is based on individual rights and justice.^5^

A review of recent studies involving students from 5 different professions, i.e., medicine, veterinary medicine, dentistry, nursing and law using the Defining Issues Test (DIT) showed that: 1. Professional education hardly encouraged the development of moral reasoning; 2) The interventions, when done properly, would increase the moral reasoning ability, 3) The moral reasoning showed the differences between subgroups, based on maturity, religion, culture and gender, however, all require further exploration; and 4) There is a correlation between moral reasoning with the clinical performance and competence.^6^^-^^8^

Personal reflection ability and moral reasoning are two qualities central to the principles of professionalism.^9^ The professionalism of medical doctors is very important in quality health services.^10^^-^^11^ Professionalism is one area of competence of medical doctors that forms the foundation of the competence of medical doctors. Doctors must demonstrate in their professional services, the highest ethical and moral standards, enriched with up-to-date knowledge and skills, and have excellent communication skills. Medical education institutions should be able to produce professional medical doctors.^12^ Professional medical doctors are not only competent, but they also must have and apply the appropriate professional values. However, medical education institutions usually concentrate on the achievement of competence and lack the attention to the development of professional values of students. Learning programs are still focused on the transfer of knowledge and skills, and learning professionalism is not a major concern.^12^

The professionalism of medical doctors not only consists of elements of competence, but also their attitude, behavior, ethics, and morals. One important part of this medical professionalism is the moral reasoning. In general, moral reasoning can be considered as an individual or collective practical reasoning about something morally important and the appropriate actions to be performed. For example, when an individual is faced with a moral question in everyday life, sometimes one reacts according to conscience or instinctively and is hesitant to consider what should be done. In addition, individuals may often experience confusion and moral conflict, so that they do not have enough moral perception.^9^

Research on the relationship between personal reflection ability with moral reasoning abilities conducted by Chalmers et al. showed that the reflection ability of medical students for one year on matters related to the treatment decreased significantly. Most medical students showed a conventional level of moral reasoning both at the beginning and the end of the year and moral reasoning scores tended to decline during the first year.^9^ The present study was conducted to determine the relationship between personal reflection ability with the moral reasoning abilities of medical students of Faculty of Medicine, Public Health, and Nursing, Universitas Gadjah Mada, Yogyakarta, Indonesia.

## Methods

### Study design and participants

This research was a cross-sectional study conducted to medical students in Year-3 at the Faculty of Medicine UGM (Batch of 2012-2013) from both the regular class (native Indonesian students) and the international class (students from various citizenship). This study was conducted after obtaining ethical approval from the Medical and Health Research Ethics Committee of the Faculty of Medicine, Public Health, and Nursing UGM-Dr Sardjito General Hospital Yogyakarta Indonesia. All 368 students, 72% (265) from the regular class and 28% (103) from the international class were asked to be willing participants. The minimal sample size was determined with a level of significance at 0.05. The minimal sample size for 368 students was 192, which consisted of 138 (72%) from the regular class and 54 (28%) from the international class.

### Data collection and data analysis

Students were invited to participate voluntarily. In the class, the researcher explained the purpose of the study, the benefits, and risks. Informed consent forms and the questionnaire were distributed to the students. The students were asked to complete and return them in class. The questionnaire was anonymized for statistical analyses by an independent research assistant. A total of 293 students (79.62%) (204 students of the regular class and 89 students of the international class) signed the informed consent forms as evidence of their agreement to participate in this research.

Personal reflection ability was measured by the Groningen Reflection Ability Scale (GRAS). The GRAS used in this study was written in Bahasa Indonesia translated by the Translation Service Unit English Department, Faculty of Cultural Science, Universitas Gadjah Mada, Yogyakarta and adapted to the Indonesian version that had been used by Rifani.^13^ The GRAS contained 23 questions to be answered with a 5-point Likert scale with 1. Strongly disagree 2. Disagree, 3. Undecided, 4. Agree, and 5. Strongly Agree. The score of some of the questions (number 3, 4, 8, 12, 17 and 21) had been negatively formulated, so it needed to be reversed (1. Strongly agree, 2. Agree, 3. Undecided, 4. Do not agree, and 5. Strongly disagree). Students were asked to choose the one answer that was most appropriate to the participant, and not consider the answer as an opinion about each statement. Each statement was independent, and not correlated with other statements. Furthermore, the scores of each question were summed to obtain a single GRAS score. The GRAS high scores indicated positive personal reflection ability, while low scores indicated negative personal reflection ability. However, there was not any specific score as the limit which indicated a good reflection ability.

Students’ moral reasoning abilities were measured by the Defining Issues Test (DIT) obtained from the Office for the Study of Ethical Development, The University of Alabama, Tuscaloosa, USA in the English version. The DIT was subsequently translated into Bahasa Indonesia, and previously given to some other students, then translated into English again and ultimately retranslated into Bahasa Indonesia for a final trial to different students before it was given to the participants. The DIT contains six stories that require the moral and ethical consideration of the participants. In the answer column of each story, the participants were asked to recommend what to do by the people mentioned in the story. Students were asked to select only the one action chosen. If students could not choose one, then they should check the option "could not decide." Each story consists of 12 questions with five possible answers (1. Great, 2. Much, 3. Some, 4. Little, 5. No). Students were asked to read each question and think about the moral and ethical considerations for each question. If the questions were considered important in making the decision, the student was asked to select "great." If the question was not important or did not make sense, they were asked to select "no." If the question was relevant but not essential, they were asked to select from "much," "some," or "little," depending on how important the consideration to the participants. Participants could select the answer "great" or other levels of importance, but there was not any specific item number which should be marked with a certain degree of importance. After the students completed all 12 questions, students were asked to choose the question that was the most important consideration. Students were asked to choose among the questions available, even if a student felt no single item was "great" in importance. The students were asked to choose only one as the most important (relating to all others), then rank the second, third, and fourth. DIT scores were then counted manually to get the Principled Morality Score (P) according to the instructions in the manual, and the means were calculated for all students, both the regular class as well as the international class. Questionnaire data could not be used when: 1. The answer did not show the real answer, 2. There was inconsistency in two stories or more, and 3. There were discrimination ratings on two stories or more. The P DIT scores were also grouped based on the scores, i.e. low, moderate, and high.^14^ Some DIT questionnaire data could not be analyzed because of incomplete data, or it had complete data, but the answer did not show the actual answer, or there were any inconsistencies in fulfilling the questionnaire. Among 204 regular class students, the data of 17 (20.99 %) female and 20 (16.26 %) male students could not be used, so that 167 data could be analyzed. Among the 89 subjects of international class, the data of 14 (40 %) female and 25 (43.82 %) male students could not be used, so that 50 data could be analyzed. The total usable data from both classes were 217 students’ complete responses.

The mean difference of GRAS scores or P DIT between the regular class and international class, and between male and female students were analyzed by T-test. The correlation between personal reflection ability and moral reasoning ability was determined by regression analysis.

## Results

### Personal reflection ability

The mean GRAS score of all students in both the regular and international classes (217 students) was 89.59 (SD=6.80) which showed a high score (GRAS score ranged 0-110). The mean GRAS score of all (167) students in the regular class was 89.19 (SD=6.47), which was not much different from the mean GRAS score 90.94 (SD=7.73) of all (50) students in the international class (t_(__217)_=1.600, p=0.153). The mean GRAS score of all (85) male students was 89.53 (SD=6.86) which was not much different from the mean GRAS score 89.64 (SD=6.79) of all (132) female students (t_(__217)_=0.113, p=0.928) (Table 1).

**Table 1:** Personal reflection ability of medical students of Faculty of Medicine Universitas Gadjah Mada Year-3 measured by The Groningen Reflection Ability Scale (GRAS), N=293

Students	GRAS Score Mean (SD, n)		
	Regular Class	International Class	All
Male	88.97 (6.00, 64)	91.23 (8.96, 21)	89.53 (6.86, 85)^ *^
Female	89.33 (6.77, 103)	90.72 (6.87, 29)	89.64 (6.79, 132)
All	89.19 (6.47, 167)	90.94 (7.73, 50)	89.59 (6.80, 217)

*T-test between male students in regular class with international class, t(85)=1.321, p=0.023

In the regular class, there was no significant difference (t_(__167)_=0.350, p=0.200) between the mean GRAS scores of 64 male 88.97 (SD=6.00) and 103 female 89.33 (SD=6.77) students. Likewise in the international class, there was no significant difference (t_(__50)_=0.230, p=0.191) between the mean GRAS scores of 21 male 91.23 (SD=8.96) and 29 female 90.72 (SD=6.87) students. The mean GRAS scores of 21 male students of the international class 91.23(SD=8.98) was slightly higher (t_(__85)_=0.321, p=0.023) than 64 male students (88.97 (SD=6.00) of the regular class. However, there was no significant difference between the mean GRAS score of 103 female students 89.33 (SD=6.77) in the regular class with 29 female students 90.72 (SD=6.87) in the international class (Table 1).

### Moral reasoning ability

The mean scores of moral reasoning ability of medical students of the Faculty of Medicine UGM calculated from P DIT scores are presented in Table 2. The mean score of P DIT of all 217 students 32.39 (SD=11.04) was between 28-41, which indicated that the score was in the moderate level. The mean of P DIT scores of 167 students in the regular class 32.58 (SD=11.18) did not significantly differ (t_(__217)_=0.458, p=0.548) from 50 students in the international class 31.77 (SD=10.61). The overall mean score of P DIT of 85 male students 29.50 (SD=10.58) did not significantly differ (t_(__217)_=3.159, p=0.537) from the mean score of P DIT of 132 female students 34.26 (SD=10.96). In the regular class, there was no significant difference (p>0.05) between the P DIT mean score of 64 male 29.18 (SD=10.35) and 103 female 34.69 (SD=11.21) students (t_(__167)_=3.178, p=0.591). In the international class, there was no significant difference (t_(__50)_=0.728, p=0.900) between the P DIT mean score of 21 male 30.47 (SD=11.46) and 29 female 32.70 (SD=10.05) students. The P DIT scores of 64 male students 29.18 (SD=10.35) in the regular class did not significantly differ (t_(85)_=0.483, p=0.988) from 21 male students in the international class 30.47 (SD=11.46), as well as between 103 female students 34.69 (SD=11.21) in the regular class and 29 female students 32.70 (SD=10.05) in the international class (t_(132)_=0.863, p=0.819).

**Table 2:** Moral reasoning ability of medical students of Faculty of Medicine Universitas Gadjah Mada Year-3 measured by The Defining Issue Test (DIT), N=293

Students	P DIT Score, Mean (SD, n)		
	Regular Class	International Class	All
Male	29.18 (10.35, 64)	30.47 (11.46, 21)	29.50 (10.58, 85)
Female	34.69 (11.21, 103)	32.70 (10.05, 29)	34.26 (10.96, 132)
All	32.58 (11.18, 167)	31.77 (10.61, 50)	32.39 (11.04, 217)

Moral reasoning ability can be classified into three groups: low (0-27), moderate (28-41), and high (42 or more).^14^ In this study, 92 (42.39%) of all 217 students showed moderate moral reasoning ability scores. Among male students of the regular class, the majority (45.31%) of students also have moderate P DIT scores, and likewise the female students (43.69%). However, it was different among international class students. The percentage of male students who have moderate P DIT score (43.69%) was equal to the percentage of students that have low P DIT score. Among female students, the majority (44.83%) have low P DIT scores. The high P DIT scores were mostly showed by female students, both in the regular class and the international class (Table 3).

### Correlation between personal reflection and moral reasoning ability

Among regular class students, personal reflection ability of 64 male students showed a positive correlation with their moral reasoning ability. However, it was not significant statistically (r=0.053, p=0.679). Personal reflection ability of 103 female students showed a positive correlation (r=0.263, p=0.07) with moral reasoning ability. However, it was not significant statistically. A total of 26.3% moral reasoning score variance of female students in the regular class can be estimated from their personal reflection ability scores.

Among the international class students, personal reflection ability of 21 male students showed a negative correlation with moral reasoning abilities, however it was not significant statistically (r=0.135, p=0.560). Personal reflection ability of 29 female students showed a negative correlation with their moral reasoning ability, however, it was not significant statistically (r=0.349, p=0.063).

Personal reflection ability scores of 167 students in the regular class showed a positive correlation with their moral reasoning ability, which was considered significant (r=0.193, p=0.012). A total of 19.3% moral reasoning ability scores variance of regular class students can be estimated from their personal reflection ability scores. In contrast, personal reflection ability scores of all 50 students in the international class showed a negative correlation with their moral reasoning ability. However, it was not significant statistically (r= 0.240, p=0.930).

Personal reflection ability scores of 85 male students showed a positive correlation (r=0.004, p=0.971) with their moral reasoning ability. However, it was not significant statistically. The personal reflection ability scores of all 132 female students showed positive correlation (r=0.133, p=0.128) with their moral reasoning ability. However, it was not significant statistically. A total of 13.3% variance of moral reasoning ability scores of female students can be estimated from their personal reflection ability scores. In general, personal reflection ability scores of all students were positively correlated (r=0.080, p=0.239) with their moral reasoning ability score. However, this correlation was not significant statistically.

## Discussion

In this study, there were no differences in any GRAS score means between the regular class students with international class students, nor for the mean GRAS scores of all male students compared to all female students. The mean GRAS scores of female students in the regular class were not much different from female students in the international class. However, the GRAS scores of male students in the international class were higher compared to male students in the regular class. This difference may be caused by the different perceptions of the experiences of each person, resulting in differences in the reflection process.

The reflection ability also varied. Many factors with various aspects affect the process of reflection. The challenging aspects of the situation also can stimulate a reflection.^4^ Results showed male students in the international class with various citizenship might be more challenged in solving challenging cases after their 3 years of medical education than male students in regular classes.

**Table 3:** Moral reasoning ability score distribution of medical students of Faculty of Medicine Universitas Gadjah Mada Year-3

Class	Sex	Moral Reasoning Ability (P DIT) n (%)			
		Low (0-27)	Moderate (28-41)	High (42 or more)	Number
Regular	M	28 (43.75)	29 (45.31)	7 (10.94)	64
	F	30 (29.13)	45 (43.69)	28 (27.18)	103
International	M	9 (42.86)	9 (42.86)	3 (14.29)	21
	F	13 (44.83)	9 (31.03)	7 (24.14)	29
Number		80 (36.87)	92 (42.39)	45 (20.74)	217

The overall mean GRAS scores of medical students of the Faculty of Medicine, Public Health and Nursing UGM from both the regular and international classes, including males and females, showed high scores. According to Aukes et al., there was not any specific score as the limit that indicates the ability of a good reflection. However, high GRAS scores indicate positive personal reflection ability, while low GRAS scores indicate negative reflection ability.^1^ The questions in the GRAS questionnaire cover the three aspects of personal reflection in the context of medical practice and medical education, i.e., self-reflection, empathy reflection, and communication reflection. Therefore, it appears that the education of students of the Faculty of Medicine UGM showed high education on their ability of these three primary reflections. These results were consistent with another study reported by Rifani, which also showed the high personal reflection ability of medical student (83.32 for the regular class and 80.86 for the international class).^13^ High personal reflection ability is considered very important for the professional development of medical students.^2^^,^^15^^,^^16^

Among all students from both the regular and international classes, male and female, there was no significant difference in the mean of P DIT scores. Their P DIT scores ranged between 28-41 which showed a moderate level.^14^ Most students (42.39%) also showed moderate moral reasoning ability scores. According to Rest, a P DIT score of 42.3 was common among students, with a mean score of 49.5 for the physicians, 52.2 for advanced students with legal expertise, and 59.8 for seminarians.^14^ Among 45 students, only six had P DIT scores equivalent to the physicians, 4 students’ scores were equivalent to advanced legal experts, and only one student’s score was equivalent to seminarians. In this study, there were only 45 (20.74%) students who showed high scores (42 or more). Students who had high scores were mostly women both in the regular class as well as in the international class. These results were consistent with results from a study by Matarazzo et al. which showed that females have higher moral reasoning and are more altruistic.^17^ In addition, women were also more concerned about the dilemmas and social problems than males.^18^

In general, the results from the present study showed that the moral reasoning ability of medical students of the Faculty of Medicine, Public Health, and Nursing, Universitas Gadjah Mada needs to be improved. The results were consistent with the study by Chalmers et al. which showed that the ability of the moral reasoning of medical students tends to decline at the end of the year.^9^ Self and Baldwin reported that there was not any difference in moral reasoning scores among four levels of a medical student during their medical education.^19^ The similar result was also obtained among veterinary medical students,^20^ while Self and Olivarez indicated the regression of moral reasoning in level 4 students due to the strong socialization effects during medical education.^21^ The study by Hren et al. also showed that there was a regression of moral reasoning during medical education.^22^ However, some studies indicate that the preferential treatment they receive will increase the moral reasoning of undergraduate and postgraduate students.^19^^-^^21^ Medical doctors must solve complex problems, and they will be expected to do their best by cooperating with the patient and the patient's family, and collaborating in multi-professional, medical professional and personal aspects while learning from their experiences.

Therefore, more efforts are needed in order to improve the moral reasoning skills of medical students. Interventions that can be done for example include designing the curriculum of medical education to be equipped with more group discussions of a variety of ethical dilemmas. Creating a more ethical environment and religious atmosphere may enhance moral reasoning ability. Moral issues will be encountered not only in conventional patient care but also in high technology patient care which is growing and changing rapidly. Moral reasoning will provide a systematic approach to assess and properly deal with the ethical issues. Although it will not provide the ultimate moral answers, however, more effort is needed to avoid bias in making decisions with relevant moral considerations and to provide guidance and help in selecting a clinical decision that is ethically responsible.^23^

High personal reflection ability scores of medical students in this study were not always accompanied by a score of high moral reasoning ability. The positive correlation between the personal reflection ability with moral reasoning ability was showed by female students’ group in the regular class, all students in the regular class, and all female students, although the correlation between their personal reflection ability with their moral reasoning ability was weak only. Among the international class students, there was a negative correlation between the ability of personal reflection with moral reasoning ability. These results were not in accordance with the previous hypothesis that the higher the ability of personal reflection, the higher the moral reasoning ability. Reflection is an important activity in the learning process, especially in clinical education that allows students to connect the knowledge they have gained with the real-life situations encountered during a clinical rotation.^3^ However, the ability of moral reasoning is not only influenced by the ability of personal reflection. Many factors affect the ability of moral reasoning such as other ethics learning in the medical curriculum,^24^ education,^25^ the socio-economic and demographic environment where they live,^26^ formal principles, rights, emotional condition, consideration in harming others, as well as other cultural and internal factors.^27^ Internal factors of people that influence decisions during interactions in the medical community will also affect student’s moral reasoning. Daily life social factors experienced by students which also contribute to the learning process can affect moral reasoning. Likewise, curriculum factors prepared for medical education will affect the ability of student moral reasoning.^19^^-^^20^

## Conclusions

The results showed that medical students of the Faculty of Medicine UGM batch of 2012/2013 have a high personal reflection ability and moderate moral reasoning ability. Personal reflection ability of all students was correlated positively with moral reasoning ability, but it was not significant statistically. The high level of personal reflection ability needs to be maintained for example by providing the appropriate learning environment and the professional behavior of lecturers, tutors, and instructors so that they stimulate the personal reflection practice. More efforts are needed to improve the moral reasoning ability of all students. Besides the addition of current medical material about morals and ethics which can be integrated into the scenarios in small group discussion, the discussion about morality and ethics can also be designed as another small group discussion with an academic supervisor at regular meetings. In addition, further study is necessary to assess other factors which influence the student’s moral reasoning ability, so that appropriate interventions can be developed according to these factors to increase their moral reasoning ability.

### Acknowledgments

The authors would like to thank Dr L.C. Aukes from the University of Groningen for the GRAS. Thanks to Mora Claramita, Efrayim Suryadi, and Ova Emilia, Sp. OG (K), for their recommendations for this research.

### Conflict of Interest

The authors declare that they have no conflict of interest.
